# Three novel *Enterobacter cloacae* bacteriophages for therapeutic use from Ghanaian natural waters

**DOI:** 10.1007/s00705-024-06081-9

**Published:** 2024-07-05

**Authors:** O. L. Lyytinen, C. Dapuliga, D. Wallinger, S. Patpatia, B. J. Audu, S. J. Kiljunen

**Affiliations:** 1 Human Microbiome Research Program (HUMI), Faculty of Medicine, University of Helsinki, Helsinki, Finland; 2 Kwame Nkrumah University of Science and Technology (KNUST), Kumasi, Ghana; 3 Division of Clinical Microbiology, HUSLAB, Helsinki University Hospital, Helsinki, Finland; 4 National Veterinary Research Institute, Vom, Nigeria

## Abstract

Infections caused by multidrug-resistant (MDR) bacteria are a growing global concern. *Enterobacter cloacae* complex (ECC) species are particularly adept at developing antibiotic resistance. Phage therapy is proposed as an alternative treatment for pathogens that no longer respond to antibiotics. Unfortunately, ECC phages are understudied when compared to phages of many other bacterial species. In this Ghanaian-Finnish study, we isolated two ECC strains from ready-to-eat food samples and three novel phages from natural waters against these strains. We sequenced the genomic DNA of the novel *Enterobacter* phages, fGh-Ecl01, fGh-Ecl02, and fGh-Ecl04, and assessed their therapeutic potential. All of the phages were found to be lytic, easy to propagate, and lacking any toxic, integrase, or antibiotic resistance genes and were thus considered suitable for therapy purposes. They all were found to be related to T4-type viruses: fGh-Ecl01 and fGh-Ecl04 to karamviruses and fGh-Ecl02 to agtreviruses. Testing of Finnish clinical ECC strains showed promising susceptibility to these novel phages. As many as 61.1% of the strains were susceptible to fGh-Ecl01 and fGh-Ecl04, and 7.4% were susceptible to fGh-Ecl02. Finally, we investigated the susceptibility of the newly isolated ECC strains to three antibiotics – meropenem, ciprofloxacin, and cefepime – in combination with the novel phages. The use of phages and antibiotics together had synergistic effects. When using an antibiotic-phage combination, even low concentrations of antibiotics fully inhibited the growth of bacteria.

**Supplementary Information:**

The online version contains supplementary material available at 10.1007/s00705-024-06081-9.

## Introduction

Infectious diseases are responsible for around 25% of deaths worldwide each year [40]. Multidrug-resistant (MDR) bacteria, which cause some of these infections, are a major global concern [19].

One option for combating infections caused by MDR bacteria is phage therapy: the use of bacteriophages, viruses that infect bacteria, to eliminate MDR pathogens [19, 27, 30, 54, 56, 59]. Antibiotic resistance is particularly problematic among Gram-negative, rod-shaped, bacteria because so few new antibiotics are being developed against these bacteria [5, 22]. One such group of Gram-negative enteric bacteria that pose a significant health threat is the family *Enterobacteriaceae*. This family includes many common pathogens such as *Escherichia*, *Salmonella*, *Klebsiella*, *Shigella*, and *Enterobacter* species. *Enterobacter cloacae* complex (ECC) species are widely found in nature but can also cause nosocomial infections such as sepsis, peritonitis, and urinary tract infections [3, 17, 36, 37]. This group also includes the ESKAPEE pathogens (*Enterococcus faecium*, *Staphylococcus aureus*, *Klebsiella pneumoniae*, *Acinetobacter baumannii*, *Pseudomonas aeruginosa*, *Enterobacter* spp., and *Escherichia coli*), all of which pose an enormous challenge to the health care system because of their broad resistance to the currently available antibiotics [12, 43].

This study was part of a larger Ghanaian initiative aiming to identify and isolate strains belonging to the family *Enterobacteriaceae* causing food-poisonings from ready-to-eat food products and to find phages against these pathogens that can be used for treatment of infections. Ready-to-eat food, also known as street food, refers to food products that are prepared, cooked, and packaged in a way that allows them to can be consumed without further cooking or preparation. While ready-to-eat foods can offer convenience, there is a potential risk of food poisoning associated with them if proper food safety practices are not followed [9]. In many African countries, including Ghana, ready-to-eat foods have been identified as common sources of food-borne illnesses [1, 9, 15, 20, 41]. In a Ghanaian study, 52 out of 60 (86.7%) ready-to-eat food samples contained higher-than-acceptable bacterial counts (> 5.00 log_10_ cfu/ml) [15]. Bacterial contamination of food can cause mild or severe diarrhea. In African children, diarrheal diseases are one of the major reasons for hospitalization, and they have been estimated to be the cause of approximately 16% of child deaths [6]. In the Ghanaian study mentioned above, ECC species were found in 6.6% of the street food samples tested [15]. These species are naturally resistant to several antibiotics, such as β-lactam antibiotics due to their chromosomally encoded β-lactamases [52], and they can easily acquire new resistance genes under conditions such as in hospital settings where antibiotics are used heavily [3, 36]. In this way, nosocomial (i.e., hospital-acquired) MDR ECC infections are constantly emerging all over the world [3, 36]. Many ECC strains have also developed resistance mechanisms against carbapenem antibiotics, which have been generally considered the last remaining resort against antibiotic-resistant Gram-negative bacteria [3, 57].

Phage therapy may be an attractive treatment option against infections caused by MDR ECC species, but the small number of characterized *Enterobacter* phages makes the treatment of ECC infections more challenging than treating many other bacterial infections. This is because, in personalized phage therapy, a specific phage or cocktail of phages is needed for each bacterial strain. By April 2024, only 98 complete genome sequences of *Enterobacter* phages were published in the GenBank database [45]. Considering that the total number of published phage genome sequences is 27,613, sequenced *Enterobacter* phages are very rare [45]. Moreover, only a fraction of these phages have been thoroughly analyzed for their suitability for phage therapy applications [31, 44, 46, 60, 62]. Notable examples include the karamviruses Enterobacter phage myPSH1140 [31] and Enterobacter phage Ec_L1 [46] and the agtreviruses Enterobacter phage EspM4VN [60] and phage vB_EclM_CIP9 [62]. Additionally, there is evidence suggesting that the *Enterobacter* phages E-2, E-3, and E-4 can effectively reduce *E. cloacae* levels in urine [44]. However, case studies utilizing phages against ECC species in patients are currently lacking in Western countries [13].

In this study, we isolated two ECC strains from Ghanaian ready-to-eat food products and also isolated three novel bacteriophages against these strains from natural waters in Ghana. The genome sequences of these three new phages, named fGh-Ecl01, fGh-Ecl02, and fGh-Ecl04, were determined and analyzed meticulously in order to assess their suitability for phage therapy purposes. In addition, we tested the host range of these phages, using a collection of Finnish clinical strains, evaluated the phylogeny of the phages, and assessed the antibiotic sensitivity of their host strains.

## Materials and methods

### Bacterial strains and culture conditions

The bacterial host strains *E. cloacae* #6957 and #6958 were isolated from ready-to-eat salads, containing onions, cabbage, lettuce, and carrots, from food vendors in Kumasi, Ghana, using a bacteriological analytical method for isolation of *E. coli* O157 [16]. Briefly, triplicate samples were mixed, and 225 ml of Maximum Recovery Diluent (Oxoid, BM0204) was added per 25 g of the mixture and vortexed for 60 s. The sample was then inoculated onto MacConkey agar (Oxoid, CM0115) and Eosine Methylene Agar (EMBA; Oxoid, CM00). Pink (MacConkey) and metallic colonies (EMBA) were picked onto Sorbitol MacConkey agar (SMA; Oxoid, CM0813) supplemented with Cefixime-Tellurite Supplement (Oxoid, SR017). Finally, colorless, non-sorbitol-fermenting colonies were further screened based on agglutination of strains containing the O157 serogroup antigen, using an *E. coli* O157 Latex Test (Oxoid, DR0620). The collection of clinical strains used for phage host range screening (Table 1) was kindly provided by the Hospital District of Helsinki and Uusimaa Laboratories (HUSLAB), Finland. Bacteria were cultivated in lysogeny broth (LB; 1% [w/v] tryptone, 0.5% [w/v] yeast extract, and 1% [w/v] NaCl). LB agar plates were supplemented with 1.0% (w/v) or 1.5% (w/v) agar (Bacto Agar, Neogen). Bacteria were cultivated overnight at 37°C unless stated otherwise.

**Table 1:** Enterobacter cloacae* and other *Enterobacteriaceae* strains used for host range screening

Bacterial strain	Source/reference	fGh-Ecl01	fGh-Ecl02	fGh-Ecl04
*Enterobacter cloacae* #6957	This study	+++ ¤	+++ ¤	+++
*Enterobacter cloacae* #6958	This study	+++	+++	+ ¤
*Enterobacter cloacae* #6947	Synlab	+++	-	+++
*Enterobacter cloacae* #6595	HUSLAB	+	-	++
*Enterobacter cloacae* #5830	HUSLAB	-	-	-
*Enterobacter cloacae* #5825	HUSLAB	+++	+++	+++
*Enterobacter cloacae* #5824	HUSLAB	-	-	-
*Enterobacter cloacae* #5823	HUSLAB	+++	-	+++
*Enterobacter cloacae* #5822	HUSLAB	-	-	+
*Enterobacter cloacae* #5821	HUSLAB	-	-	-
*Enterobacter cloacae* #5820	HUSLAB	+++	-	++
*Enterobacter cloacae* #5819	HUSLAB	+++	-	+++
*Enterobacter cloacae* #5818	HUSLAB	-	-	-
*Enterobacter cloacae* #5817	HUSLAB	+++	-	+++
*Enterobacter cloacae* #5816	HUSLAB	+++	-	+++
*Enterobacter cloacae* #5815	HUSLAB	+++	-	+++
*Enterobacter cloacae* #5814	HUSLAB	-	-	-
*Enterobacter cloacae* #5813	HUSLAB	+++	-	+++
*Enterobacter cloacae* #5812	HUSLAB	-	-	-
*Enterobacter cloacae* #5811	HUSLAB	+++	-	+++
*Enterobacter cloacae* #5810	HUSLAB	+++	-	++
*Enterobacter cloacae* #5809	HUSLAB	+++	-	+++
*Enterobacter cloacae* #5808	HUSLAB	+++	+++	+++
*Enterobacter cloacae* #5807	HUSLAB	-	-	-
*Enterobacter cloacae* #5806	HUSLAB	+	-	-
*Enterobacter cloacae* #5805	HUSLAB	+++	-	++
*Enterobacter cloacae* #5804	HUSLAB	-	-	-
*Enterobacter cloacae* #5803	HUSLAB	+++	-	+++
*Enterobacter cloacae* #5802	HUSLAB	+++	-	+++
*Enterobacter cloacae* #5738	HUSLAB	-	-	-
*Enterobacter cloacae* #5737	HUSLAB	+++	-	+++
*Enterobacter cloacae* #5736	HUSLAB	-	-	-
*Enterobacter cloacae* #5735	HUSLAB	+++	-	+++
*Enterobacter cloacae* #5734	HUSLAB	+++	-	+++
*Enterobacter cloacae* #5733	HUSLAB	+++	-	+++
*Enterobacter cloacae* #5708	HUSLAB	++	-	++
*Enterobacter cloacae* #5666	HUSLAB	-	-	-
*Enterobacter cloacae* #5665	HUSLAB	-	-	-
*Enterobacter cloacae* #5664	HUSLAB	+++	+++	++
*Enterobacter cloacae* #5663	HUSLAB	+++	-	+++
*Enterobacter cloacae* #5662	HUSLAB	+++	+++	++
*Enterobacter cloacae* #5661	HUSLAB	++	-	+++
*Enterobacter cloacae* #5660	HUSLAB	+++	-	+++
*Enterobacter cloacae* #5659	HUSLAB	-	-	-
*Enterobacter cloacae* #5658	HUSLAB	-	-	-
*Enterobacter cloacae* #5562	HUSLAB	-	-	-
*Enterobacter cloacae* #5561	HUSLAB	-	-	-
*Enterobacter cloacae* #5557	HUSLAB	-	-	-
*Enterobacter cloacae* #5556	HUSLAB	++	-	+++
*Enterobacter cloacae* #5554	HUSLAB	-	-	-
*Enterobacter cloacae* #5544	HUSLAB	+	-	++
*Enterobacter cloacae* #5541	HUSLAB	+++	-	+++
*Enterobacter cloacae* #5534	HUSLAB	+++	-	+++
*Enterobacter cloacae* #5532	HUSLAB	+++	-	+++
*Enterobacter cloacae* #5508	HUSLAB	-	-	-
*Enterobacter cloacae* #804	MBL Turku	-	-	-
*Escherichia coli* #7141	Fimlab	-	-	-
*Escherichia coli* #6580 (103264)	DSMZ	-	-	-
*Escherichia coli* #6127	HUSLAB	-	-	-
*Escherichia coli* #5710 ([BL21] Star DE3)	Invitrogen	-	-	-
*Escherichia coli* #2228 (XL-1[pQE32]) 33323	Qiagen	-	-	-
*Escherichia coli* #1624 (XL)	Stratagene	-	-	-
*Escherichia coli* #1313 (Q359; DSM 6197)	DSMZ	-	-	-
*Escherichia coli* #833	MBL Oulu	-	-	-
*Escherichia coli* #807	MBL Turku	-	-	-
*Escherichia coli* #253 (K12, C600 su, lambda CI857)	[55]	-	-	-
*Klebsiella aerogenes* #6737	HUSLAB	-	-	-
*Klebsiella pneumonia* #6322 (DSM681)	DSMZ	-	-	-
*Klebsiella pneumonia* #6039	HUSLAB	-	-	-
*Klebsiella pneumonia* #5749	HUSLAB	-	-	-
*Klebsiella pneumonia* #5529	HUSLAB	-	-	-
*Klebsiella pneumonia* #5518	HUSLAB	-	-	-
*Yersinia enterocolitica* #564 (O:8)	MBL Oulu	-	-	-
*Yersinia enterocolitica* #443 (O:3)	MBL Oulu	-	-	-
*Yersinia enterocolitica* #193 (O:3)	KTL Turku	-	-	-
*Yersinia pseudotuberculosis* #6582	[47]	-	-	-

The Ghanaian isolation host strain of each bacteriophage is indicated by a symbol ¤

+++, strong infection, OD_600nm_ reduction >80%* after 5 h

++, medium infection, OD_600nm_ reduction 50% – 80%* after 5 h

+, weak infection, OD_600nm_ reduction 30% – 50%* after 5 h

- , no infection, OD_600nm_ reduction <30%* after 5 h

* In this table, *Enterobacter cloacae* refers to *Enterobacter cloacae* complex (ECC) species.

DSMZ: Leibniz Institute DSMZ-German Collection of Microorganisms and Cell Cultures GmbH, Germany

Fimlab: Fimlab laboratoriot Oy, Tampere, Finland

HUSLAB: The Hospital District of Helsinki and Uusimaa Laboratories, Finland

KTL Oulu: National Public Health Institute, Oulu, Finland

MBL Oulu: Faculty of Biochemistry and Molecular Medicine, University of Oulu, Oulu, Finland

MBL Turku: Department of Medical Microbiology, University of Turku, Finland

Synlab: Synlab Oy, Finland

## Bacterial host strain identification

The genomic deoxyribonucleic acids (DNAs) of the new host strains #6957 and #6958 were extracted using a ZR Genomic DNA Miniprep Kit (ZYMO Research Corp. USA) according to the manufacturer’s instructions. The preliminary screening for family *Enterobacteriaceae* strains was done using polymerase chain reaction (PCR) with the primers E16S-a (5’-CCCCCTGGACGAAGACTGAC) and E16S-b (5’-ACCGCTGGCAACAAAGGATA), which were originally designed to target *E. coli* 16S ribosomal ribonucleic acid (RNA) [61, 63]. The final confirmation of the host strains was performed using a matrix-assisted laser desorption/ionization coupled with time-of-flight (MALDI-TOF) mass spectrometer (VITEK MS, BioMérieux, France) at the Division of Clinical Microbiology, HUSLAB, Helsinki, Finland.

## Basic phage methods

Bacteriophage lysates were produced either from liquid cultures or semi-confluent plates, and the titer of each phage lysate was determined using double-layer plaque assay [48]. LB soft agar medium was solidified with either 0.4% or 0.7% agar (w/v; Bactoagar, Neogen).

## New bacteriophage isolation

Phages fGh-Ecl01 and fGh-Ecl02 were isolated from a vegetable farm irrigation pond on the Kwame Nkrumah University of Science and Technology (KNUST) campus (Kumasi, Ghana) using *E. cloacae* strain #6957 as the host bacterium, and phage fGh-Ecl04 was isolated from the River Wiwi (Kumasi, Ghana) using *E. cloacae* #6958 as the host. Ten-ml water samples were processed as described previously by Kropinski *et al*. [26]. Briefly, debris was removed by centrifugation (4200 *g*, 10 min) and the supernatant was filtered using a 0.22-µm polyether sulfone (PES) filter (All Pure Biotech). The filtrate was mixed with 10 ml of 2x tryptone soya broth (CM0129, Oxoid) containing 2 mM CaCl_2_ and 50 µl of a 1:200 dilution of an overnight-grown culture of the host bacterium and incubated for 48 h with shaking at 50 rpm. The culture was clarified by centrifugation (7170 *g*, 15 min), and the supernatant was filtered as above. Finally, a 10-µl drop of filtrate was applied onto a lawn of host bacteria growing on an LB plate. Individual plaques were purified three times using a serial dilution method described earlier by Kropinski *et al*. [26].

## Genomic DNA isolation and sequencing

The genomic DNA of fGh-Ecl04 was isolated from a fresh phage lysate using a Phage DNA Isolation Kit (Norgen Biotek), and the genomic DNA of fGh-Ecl01 and Ecl02 was isolated using traditional phenol-chloroform extraction followed by ethanol precipitation [48]. Briefly, the fresh phage lysate was treated with DNase (0.00325 U/µl) and RNase (0.01 µg/µl) at 37°C for 30 min, and then with 18.1 mM ethylenediaminetetraacetic acid (EDTA;), protease K (54.3 µg/ml), and sodium dodecyl sulfate (SDS; 4.5 mg/ml [w/v]) at 56°C for 60 min. The DNA was extracted with one volume of phenol and one volume of chloroform and then precipitated with 0.1 volume of 3 M NaOAc (pH 7.0) and two volumes of absolute ethanol. Illumina next-generation sequencing of the phage genomes was performed at Novogene, United Kingdom (https://www.novogene.com/eu-en/).

## Phage genome sequence assembly and analysis

The genome sequences of phages fGh-Ecl01, fGh-Ecl02, and fGh-Ecl04 were assembled *de novo* using the A5 (Andrew And Aaron’s Awesome Assembly) pipeline [10] with a subset of 100,000 out of 2,852,435, 4,179,013, and 4,302,692 original 150-bp reads, respectively. The assemblies were verified by mapping all the original reads to the newly formed contigs using Geneious Prime 2022.0.2. Preliminary annotation of the phage genomes was done using RAST (Rapid Annotation using Subsystem Technology; [4]), and the final annotation was done manually using Geneious Prime 2022.0.2., protein BLAST (Basic Local Alignment Search Tool; BLASTp [24]), and tRNAscan-SE version 2.0 [8]. The presence of putative antibiotic resistance and virulence genes was analyzed using CARD (Comprehensive Antibiotic Resistance Database; [32]), Resistance Gene Finder (RGI; [2]), and VirulenceFinder-2.0 [23].

## Phylogenetic analysis

The genome sequences of the three new phages were compared to other phage sequences using nucleotide BLAST (BLASTn; National Center for Biotechnology Information [NCBI]; search issued on 22 June, 2022) with standard settings [24]. Complete genome sequences with at least 80% query coverage and 90% identity were chosen for phylogenetic analysis using VICTOR (Virus Classification and Tree Building Online Resource; https://victor.dsmz.de) [33, 34] and genomic identity heat map analysis using VirClust (http://rhea.icbm.uni-oldenburg.de/virclust/) [38, 39], both with standard settings. For the phylogenetic comparison of the host-recognition proteins, distal tail fiber subunit protein sequences for fGh-Ecl01 and fGh-Ecl04 and receptor-binding spike proteins sequences for fGh-Ecl02 were chosen. All pairwise comparisons of complete genome nucleotide sequences were performed using VICTOR, by the genome-BLAST distance phylogeny (GBDP) method. The host-recognition proteins were compared using the VICTOR single-gene phylogeny server [35]. Both phylogenetic trees were rooted at the midpoint [14] and visualized using Interactive Tree of Life (iTOL) [29].

## Nucleotide sequences

The annotated genomic nucleotide sequences of the new bacteriophages were deposited in the GenBank database (NCBI) with the accession numbers ON212265 (fGh-Ecl01), ON212266 (fGh-Ecl02), and ON212267 (fGh-Ecl04).

## Host range screening and antibiotic sensitivity

Host range screening and antibiotic susceptibility analysis were performed using a previously described liquid-based protocol [28] with conditions optimized for *E. cloacae* [42]. Briefly, 190 µl of 1:200-diluted overnight host culture was mixed with 10 µl of phage lysate (10^9^ PFU/ml), resulting in a multiplicity of infection (MOI) of approximately 1. When conducting antibiotic sensitivity tests, two different concentrations of three different antibiotics were added to the growth medium: a high concentration of 60 µg/ml for all antibiotics and a low concentration of either 2 µg/ml for meropenem or 1 µg/ml for ciprofloxacin and cefepime. Assays were performed in triplicate for each host-phage (host range screening) and host-phage-antibiotic (sensitivity tests) combination. The optical density (OD_600_) was measured every 30 min, using Bioscreen FP-1100-C (Growth Curves Ab Ltd.) with continuous shaking at 37°C for 5 h for host range screening and for 15 h for antibiotic sensitivity experiments. For each time point, the average OD_600_ value and standard deviation were calculated using Microsoft Office Excel 2016 (Microsoft). Graphical representations were made using the OriginPro 2022b program (OriginLab). In host range screening, a strong phage infection was considered to have occurred if the absorbance of the culture was reduced by >80% relative to the control without phage after 5 hours. The phage infection was considered to be of medium strength if the growth reduction was 50–80%, weak if the growth reduction was 30–50%, and negative (no infection) if the growth inhibition was <30% of the control.

## Results

### Host strains belong to the Enterobacter cloacae complex (ECC) species

The initial screening of the isolated host strains #6957 and #6958 suggested that they belong to the species *E. coli*, since amplifying their isolated genomic DNA with primers specific for the 16S RNA of *E. coli* resulted in clear amplification products with electrophoretic mobility corresponding to that of the expected product with a length of 401 bp (Supplementary Fig. S1). However, MALDI-TOF analysis revealed that they, in fact, both belonged to the species *E. cloacae* or *E. asburiae*, with 50% probability of each, and should thus be considered members of the *E. cloacae* species complex (ECC).

### Biological and genomic features of the newly isolated bacteriophages

All three of the new phages, fGh-Ecl01, fGh-Ecl02, and fGh-Ecl04, produced clear plaques with halos of different sizes (Supplementary Fig. S2). Genome sequencing revealed that the genomes of fGh-Ecl01 and fGh-Ecl04 are 171.7 and 172.4 kbp in length, respectively, and both have a GC content of 39.8% (Table 2). Phage fGh-Ecl01 has 289 protein-coding genes, and fGh-Ecl04 has 288, and they both have 19 transfer RNA (tRNA) genes (Table 2). Phage fGh-Ecl02 differs from these two phages, with a genome size of 153.3 kbp, 49.2% GC content, 192 protein-coding genes, and four tRNA genes (Table 2). The genomes of the new phages were not predicted to contain antibiotic resistance or virulence genes, according to analysis using CARD, Resistance Gene Finder [2], and Virulence Gene Finder [23] (data not shown). In addition, no genes coding for integrase or other proteins associated with a lysogenic cycle were identified in the genomes (data not shown).

**Table 2:** Phage genome data

Phage	Accession number	Size, base pairs	GC-content, %	Protein-coding genes	tRNA genes	Coverage, %*	Identity, %*	
fGh-Ecl01	ON212265	171 663	39.8	289	19	100	100	
fGh-Ecl02	ON212266	153 282	49.2	192	4	0.4	66.43	
fGh-Ecl04	ON212267	172 419	39.8	288	19	100	98.96	

*Compared to the full-length fGh-Ecl01 genomic sequence

### Phylogenetic association of fGh-Ecl01 and fGh-Ecl04 with karamviruses and fGh-Ecl02 with agtreviruses

**Fig. 1:**
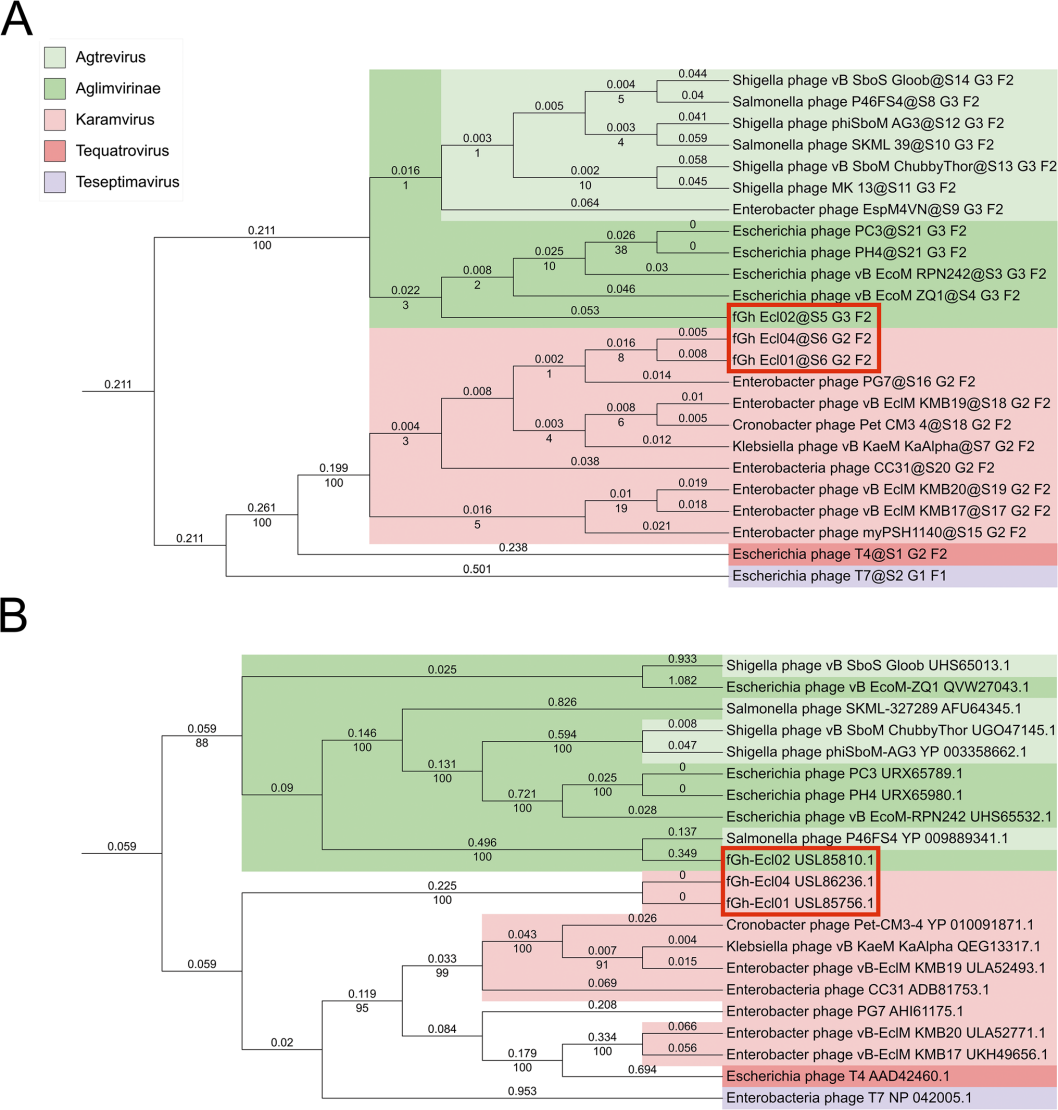
Phylogenetic trees of the new *Enterobacter* phages and their closest relatives. These trees represent the phylogenetic relationships of (**A**) the whole-genome nucleotide sequences and (**B**) the amino acid sequences of the host recognition proteins of the *Enterobacter* phages fGh-Ecl01, fGh-Ecl02, and fGh-Ecl04 (indicated by red rectangles) to their closest relatives (determined using BLASTn), Escherichia phage T4, and Enterobacteria phage T7. The analysis was done using VICTOR [34], and the trees were visualized using iTOL [29]. Both trees were rooted at the midpoint. The numbers above the branches represent the branch lengths, and those below the branches are the GBDP pseudo-bootstrap support values from 100 replications. Color-coding: light green, *Agtrevirus*; dark green, *Aglimvirinae*; light red, *Karamvirus*; dark red, *Tequatrovirus*; violet, *Teseptimavirus*

According to a BLASTn search analysis using whole-genome sequences, the closest relatives of phages fGh-Ecl01 (ON212265) and fGh-Ecl04 (ON212267) belong to the genus *Karamvirus* (subfamily *Tevenvirinae,* family *Straboviridae,* class *Caudoviricetes* [25]; Supplementary Table S1), whereas those of phage fGh-Ecl02 (ON212266) belong to the genus *Agtrevirus* (subfamily *Aglimvirinae*, family *Ackermannviridae*, class *Caudoviricetes* [25]; Supplementary Table S1). Nevertheless, a genome-wide phylogenetic analysis using VICTOR [34] assigned all three of these phages to the same family cluster (F2) with *Escherichia* phage T4 (Fig. 1A). In the sublevel clustering, VICTOR grouped fGh-Ecl01 and fGh-Ecl04 together with *Enterobacter* phages and *Escherichia* phage T4 (G2), categorizing fGh-Ecl01 and fGh-Ecl04 as members of the same species (S6; Fig 1A), whereas fGh-Ecl02 was associated with *E. coli*, *Salmonella*, and *Shigella* phages (G3; Fig. 1A). When analyzing the relationships among these phages using the VirClust sequence identity heat map tool, the genomes of fGh-Ecl01 and fGh-Ecl04 had the highest sequence identity (97%) to that of a member of the species *Karamvirus pg7* (formerly *Enterobacter virus PG7*), which also belongs to the genus *Karamvirus* (Supplementary Fig. S3). The genome sequence of fGh-Ecl02 had the highest similarity to those of *Escherichia* phage vB EcoM RPN242 (92% identity) and *Escherichia* phage vB EcoM ZQ1 (91% identity), *Escherichia* phage PC3 (91% identity), and *Escherichia* phage PH4 (91% identity) (Supplementary Fig. S3), all classified within the subfamily *Aglimvirinae* of the family *Ackermannviridae*.

When the predicted amino acid sequences of the distal tail fiber subunits (fGh-Ecl01 and fGh-Ecl04) and spike proteins (fGh-Ecl02) were used for phylogenetic analysis, the resulting phylogenetic tree (Fig. 1B) was mostly similar to the one obtained using whole-genome sequences (Fig. 1A). Only viruses assigned to the genus *Agtrevirus* were more dispersed (Fig. 1B). Interestingly, the spike protein of fGh-Ecl02 clusters with that of *Salmonella* phage P46FS4, whereas, in the whole-genome analysis, these phages cluster differently (Fig. 1A and B). The size of the P46FS4 spike protein is less than one fourth of that of fGh-Ecl02 (159 aa. vs. 700 aa., Supplementary Table S2) and it is the smallest spike protein included in this analysis (Fig. 1B; Supplementary Table S2). The distal tail fiber proteins of fGh-Ecl01 and fGh-Ecl04 are identical to each other, and they cluster with *Enterobacter* phage tail fiber proteins (Fig. 1A and B).

### FGh-Ecl01 and fGh-Ecl04 have a broader host range than fGh-Ecl02

The newly isolated *Enterobacter* phages were tested against 55 clinical ECC strains isolated from Finnish patients (Table 1). Phages fGh-Ecl01, fGh-Ecl02, and fGh-Ecl04 strongly infected 50.0%, 7.4%, and 42.6% of the tested clinical strains, respectively (Table 1). If medium and weak infections were also included, this percentage rose to 61.1% for both fGh-Ecl01 and fGh-Ecl04 but remained the same (7.4%) for fGh-Ecl02 (Table 1). Strains representing other species of the family *Enterobacteriaceae*, including *E. coli* (n = 10), *Klebsiella* spp. (n = 6), and *Yersinia* spp. (n = 4), were all resistant to the tested phages (Table 1).

### Phages and antibiotics show a synergistic effect against Enterobacter strains

**Fig. 2:**
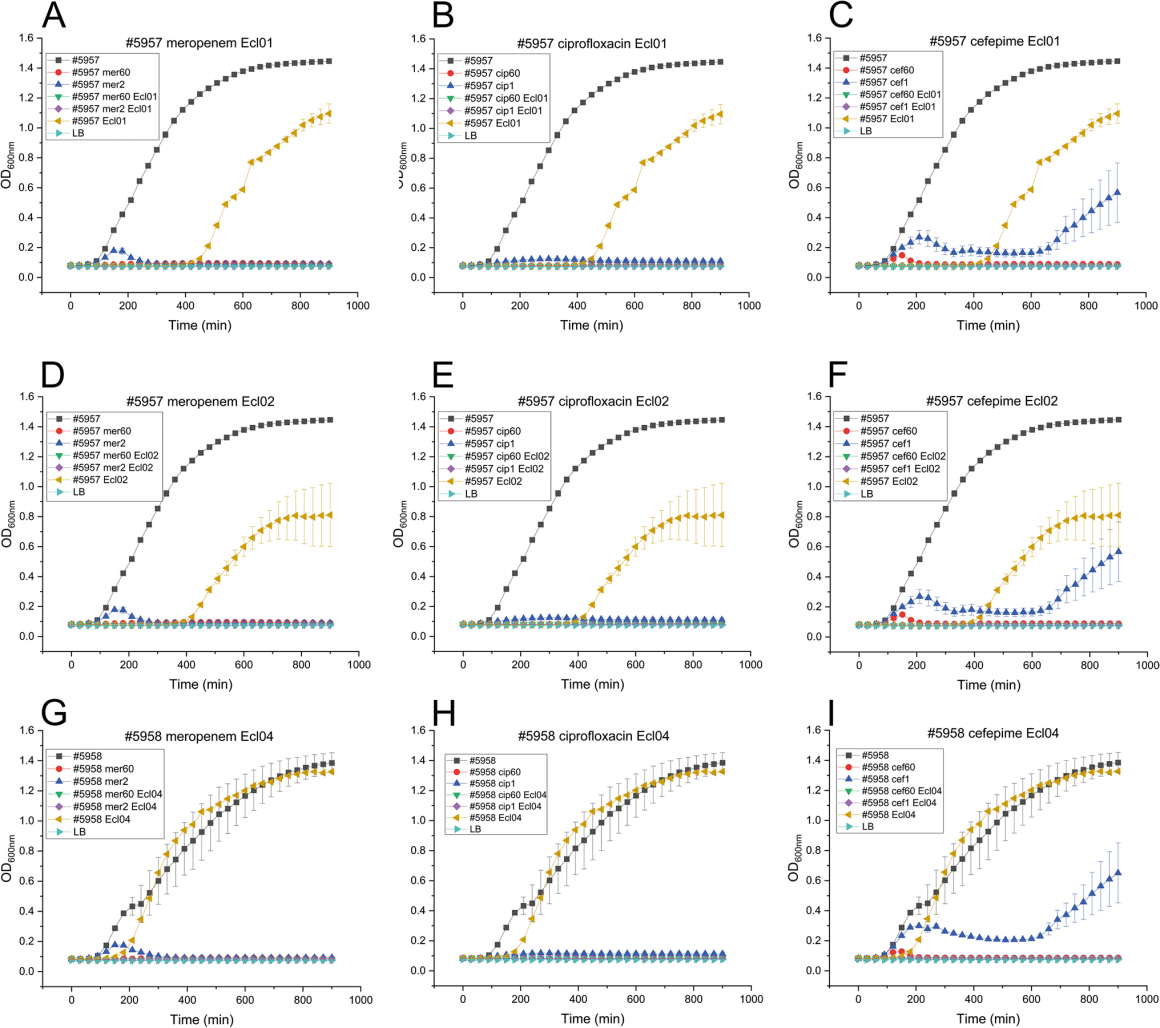
Susceptibility of the new *Enterobacter cloacae* strains to antibiotics and phages. We tested the susceptibility of the two newly isolated *E. cloacae* strains, #5957 (A-F) and #5958 (G-I), to high (60 µg/ml) and low (1-2 µg/ml) concentrations of meropenem (A, D, and G; low 2 µg/ml), ciprofloxacin (B , E, and H; low 1 µg/ml), and cefepime (C, F, and I; low 1 µg/ml), as well as to the specific phages fGh-Ecl01 (A, B, and C) and fGh-Ecl02 (D, E, and F) for #5957 and fGh-Ecl04 for #5958 (G, H, and I). The optical density (OD_600nm_; *y*-axis) of the culture was measured every hour for 15 hours (900 minutes; *x*-axis)

Finally, we tested the sensitivity of the newly isolated *E. cloacae* strains #6957 and #6958 to the three antibiotics commonly used for ECC infections – meropenem, ciprofloxacin, and cefepime – and evaluated the potential synergy between phage and antibiotic usage (Fig. 2). At the high concentration (60 µg/ml) of all antibiotics, host growth was completely inhibited (Fig. 2A–F). At a low concentration of meropenem, (2 µg/ml) both strains exhibited weak growth, but after 250 minutes, their growth ceased completely (Fig. 2A and D). At a low concentration of ciprofloxacin (1 µg/ml), host growth was consistently weak throughout the experiment (Fig. 2B and E). Furthermore, when subjected to a low cefepime concentration of 1 µg/ml, both strains initially exhibited weak growth (Fig. 2C and F), but after 600 minutes, there was a notable increase in growth (Fig. 2C and F). With all ECC strain and phage combinations, bacterial growth was initially inhibited but recovered after approximately 400 minutes for the #6957+fGh-Ecl01 and #6957+Ecl02 combinations (Fig. 2A, B, and C), and after 200 minutes for #6958+fGh-Ecl04 (Fig. 2D, E, and F). In all of the reactions, the phage-antibiotic combination completely inhibited bacterial growth (Fig. 2A–F).

## Discussion

Diarrheal diseases are the leading cause of hospitalization among African children, accounting for approximately 16% of child deaths [6]. Bacterial contamination of food can cause mild or severe diarrhea. This study constituted a component of a broader Ghanaian initiative aiming to identify and isolate strains belonging to the family *Enterobacteriaceae* causing foodborne illnesses from ready-to-eat food products and to find phages against these pathogenic species that can be used to treat infections. In this study, we successfully isolated three previously undiscovered bacteriophages with their respective host bacteria and assessed their potential application in phage therapy.

Although MDR pathogens are one of the major threats of our time and the rate of new antibiotic discovery has decreased [5], we are not totally left without solutions. Phage therapy has been proposed to alleviate the antibiotic resistance problem, but it brings challenges of its own [19, 27, 30, 54, 56, 59]. Phage therapy against *Enterobacteriaceae* species, such as *E. coli*, *Shigella* spp., and *Klebsiella pneumoniae*, has been investigated in multiple studies, both as prophylaxis and as treatment in humans [7, 18, 50, 51]. However, there is still a lack of case studies using phages against ECC species in patients in Western countries [13], and phages against ECC species have been isolated and thoroughly studied than against other *Enterobacteriaceae* species. To effectively employ phage therapy against infections caused by ECC species, it is imperative to discover and thoroughly characterize novel phages targeting these species.

In this study, we isolated two ECC strains, #6957 and #6958, from street food samples. The initial screening indicated that these strains belonged to the species *E. coli* (Supplementary Fig. S1), but the MALDI-TOF analysis revealed them to be ECC species (3.1). This underscores how closely related members of the genera *Escherichia* and *Enterobacter* are and the importance of using several different methods for identification of members of the family *Enterobacteriaceae*. Following the isolation of bacterial host strains, we isolated three new *Enterobacter* phages against these strains from natural waters: fGh-Ecl01, fGh-Ecl02, and fGh-Ecl04. Genome sequence analysis showed that all three phages belong to the class *Caudoviricetes* (Supplementary Table S1, Fig.1, and Supplementary Fig. S3). Both fGh-Ecl01 and fGh-Ecl04 have ~172-kbp genomes (Table 2) and their closest relatives are members of the genus *Karamvirus* (subfamily *Tevenvirinae*, family *Straboviridae*), whereas fGh-Ecl02 has a ~153-kbp genome (Table 2), and its closest relatives are members of the genus *Agtrevirus* (subfamily *Aglimvirinae*, family *Ackermannviridae*). Interestingly, although phages fGh-Ecl01 and fGh-Ecl04 have genomes that are only ~19 kbp larger than that of fGh-Ecl02, they possess a significantly larger number of protein coding genes (97 and 96, respectively). This is largely attributable to the presence of numerous small hypothetical protein-coding genes within the genomes of fGh-Ecl01 and fGh-Ecl04.

According to the guidelines of the International Committee on Taxonomy of Viruses (ICTV), bacteriophages are considered to belong to the same species if their nucleic acid sequences are >95% identical [38]. The genomes of phages fGh-Ecl01 and fGh-Ecl04 are 99% identical to each other (Supplementary Fig. S3) and thus should be considered members of the same species. Indeed, they have a very similar host range, despite a few exceptions, such as *E. cloacae* #5806 and #5822 (Table 1). Additionally, both of these phages share 97% genetic identity to Enterobacter phage PG7 (NC_023561.1), a relatively recently isolated *Enterobacter* phage that also resembles Klebsiella phage vB_KaeM_KaAlpha. Interestingly, fGh-Ecl02 is only 92% identical to its closest relative, *Escherichia* phage vB_EcoM-RPN242 [21], which is an agtrevirus with a 154.8-kb genome. Thus, fGh-Ecl02 can be considered a member of an entirely new phage species (Supplementary Fig. S3), whereas fGh-Ecl01 and fGh-Ecl04 belong to the same species as the already known *Enterobacter* phage PG7 (NC_023561.1).

For host recognition, different phages use different receptor-binding proteins. For instance, T4-like phages have long tail fibers, which are made up of multiple subunits, with the distal subunit (the one located farthest from phage’s head) being responsible for recognition and attachment to the host. Phages fGh-Ecl01 and fGh-Ecl04 also have 100% identical long tail fiber distal subunits (Fig. 1B, Supplementary Table S2). Interestingly, they nonetheless still exhibit slightly different host ranges (Table 1). For example, fGh-Ecl04 only weakly infects its isolation strain #5958, whereas fGh-Ecl01 infects the same strain strongly (Table 1). Although the receptor-binding protein is the major component determining the host range of a phage, there are other factors that can also influence the host range, such as factors that affect protein synthesis [11].

There are several requirements that bacteriophages must fulfill in order to be considered potential therapeutic phages [58]. Firstly, a therapeutic phage must be lytic, meaning it follows a lytic life cycle and lacks integrase and other genes necessary for the lysogenic cycle. Secondly, a therapeutic phage should be easily propagated in liquid or on plates. Also, the genomes of therapeutic phages must not contain any toxic or antibiotic resistance genes that could be transferred to pathogenic or symbiotic bacteria in the patient. In addition, a low propensity for development of phage resistance in the host strains is desirable. Phage resistance refers to the ability of the host to evade or defend against phage infection over time [53]. In this study, all three novel phage isolates fulfill the first three criteria. They all are T4-type phages, which, in previous studies, have not caused any side effects in children or adults [49, 51]. Phages fGh-Ecl01 and fGh-Ecl04 have a relatively broad host range, infecting up to 60% of the clinical strains tested here (Table 1). Phage fGh-Ecl02 has a relatively narrow host range, infecting only 7.4% of the tested strains, but one should remember that the phages in this study were isolated in Ghana, whereas and the tested strains were clinical strains from Finland. In this context, even the 7.4% infection rate appears remarkable, not to mention the 60% rate of fGh-Ecl01 and fGh-Ecl04. However, it should also be pointed out that the actual host specificity of these phages remains to be elucidated, because except for the MALDI-TOF analysis, the diversity of the tested strains has not been investigated. Regarding phage resistance, resistant strains emerged after 450, 400, and 200 minutes for the combinations of #6957 Ecl01, #6957 Ecl02, and #5958 Ecl04, respectively (Fig. 2). This suggests that these phages are not ideal for use as standalone agents in phage therapy but might be useful in combination therapies such as cocktails or in conjunction with antibiotics.

To investigate the synergic effects of phages and antibiotics, we tested the susceptibility of the Ghanaian ECC strains against the new phages and the antibiotics most commonly used in the treatment of ECC infections: meropenem, ciprofloxacin, and cefepime (Fig. 2). The data clearly demonstrate the efficiency of the concurrent use of phages and antibiotics (Fig. 2). In all cases, the combination of the phage and the lowest antibiotic concentration substantially inhibited the growth of the ECC strain (Fig. 2). There is an urgent need to reduce the use of antibiotics in healthcare to prevent the development of antibiotic resistance and minimize potential side effects for patients. Therefore, all three novel *Enterobacter* phages constitute a valuable addition to the therapeutic phage repository.

While phages possess remarkable potential as innovative therapeutic components, substantial efforts must be undertaken before their integration into the clinician's everyday toolkit is feasible. The community must engage in revising legislation and regulations to accommodate the unique nature of these biological entities. While there is considerable evidence of the safety of phages [49, 51], establishing their efficacy requires additional evidence through comprehensive clinical studies.

### Supplementary Information

Below is the link to the electronic supplementary material.

Supplementary file1 (PDF 1386 KB)

Supplementary file2 (PDF 1879 KB)

Supplementary file3 (PDF 1361 KB)

Supplementary file4 (PDF 20 KB)

Supplementary file5 (PDF 10 KB)

## Data Availability

The complete genome sequences of bacteriophages fGh-Ecl01, fGh-Ecl02, and fGh-Ecl04 have been deposited in the GenBank database with the accession numbers ON212265, ON212266, and ON212267, respectively.
