# Supplementary material for: Three novel Enterobacter cloacae bacteriophages for therapeutic use from Ghanaian natural waters
Source: Arch Virol. 2024 Jul 5;169(8):156. doi: 10.1007/s00705-024-06081-9 (PMC11226500; doi:10.1007/s00705-024-06081-9)
Supplement: Supplementary file 1 — Supplementary file1 (PDF 1386 KB) [file 705_2024_6081_MOESM1_ESM.pdf]

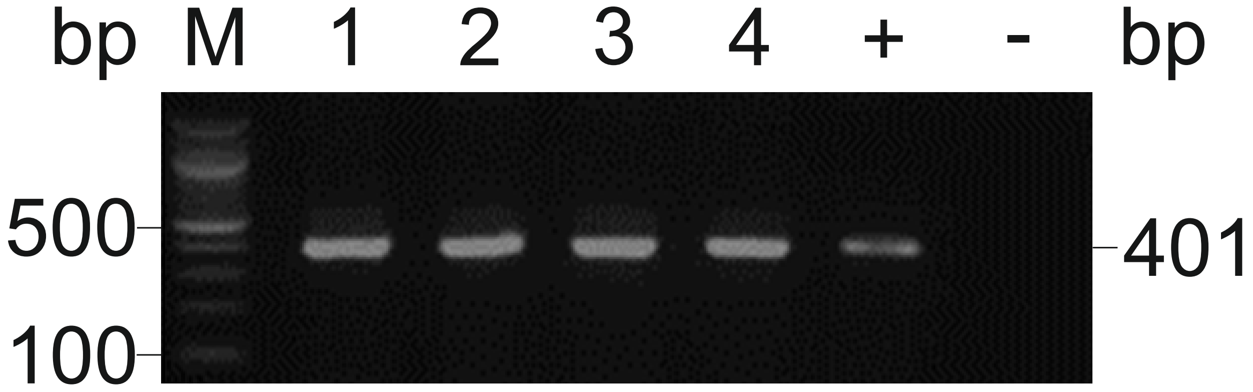

Figure S1. Preliminary PCR screening of ready-to-eat salad samples 1 - 4 for *Enterobacteriaceae* family strains M: DNA molecular weight marker, bp: base pairs,+: positive control, -: negative control.
