# Supplementary figures and images for: Three novel Enterobacter cloacae bacteriophages for therapeutic use from Ghanaian natural waters
Source: Arch Virol. 2024 Jul 5;169(8):156. doi: 10.1007/s00705-024-06081-9 (PMC11226500; doi:10.1007/s00705-024-06081-9)

A

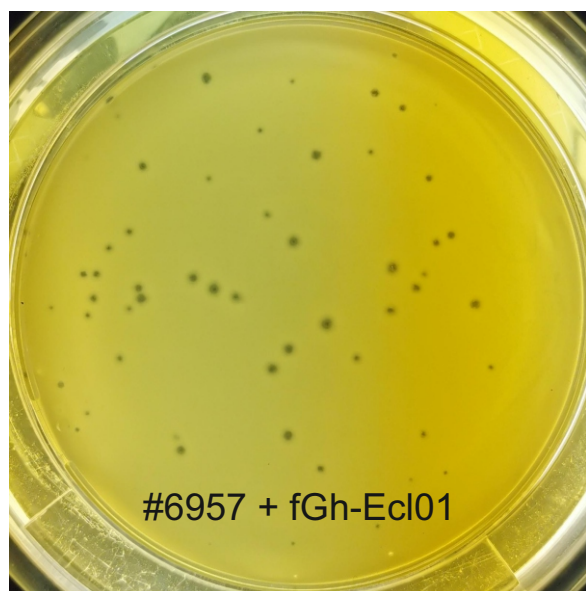

B

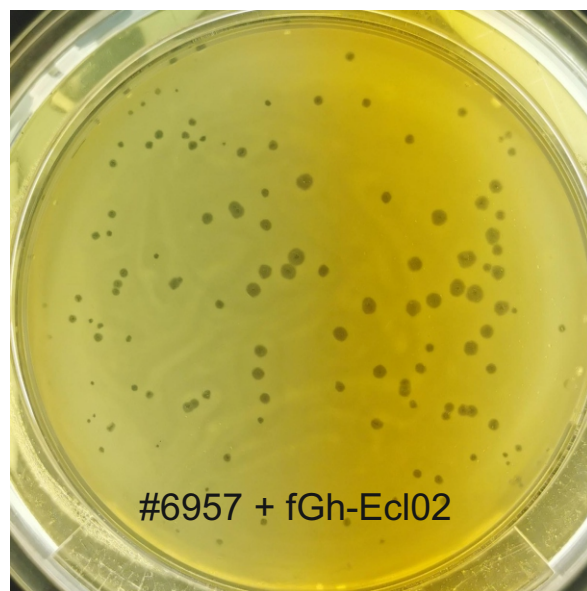

C

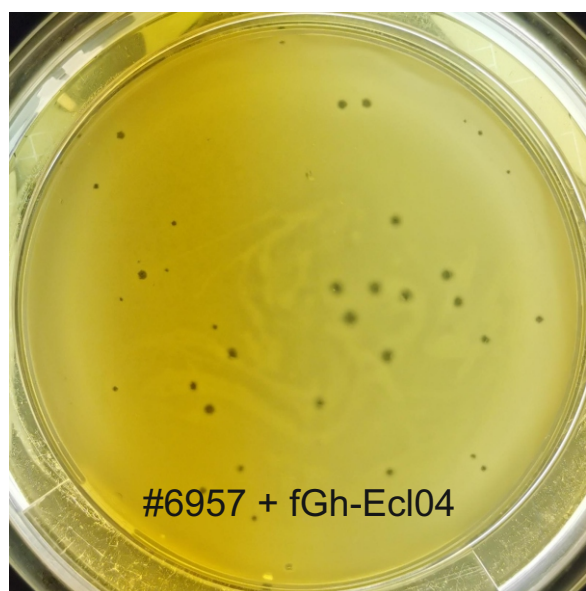

D

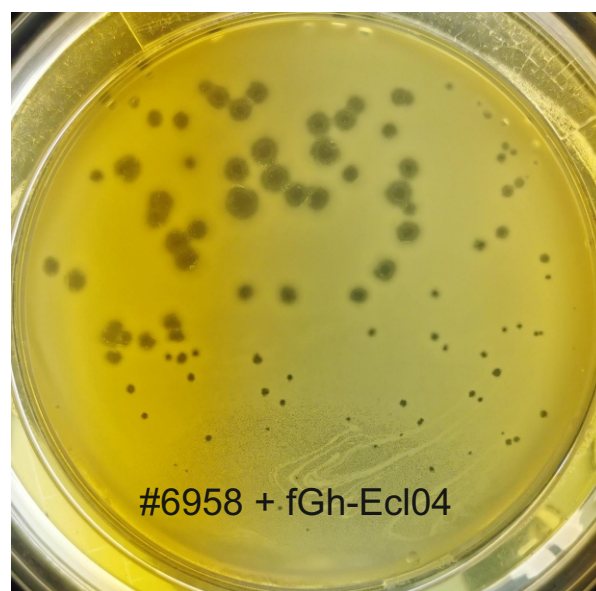

Supplement: Supplementary file 2 — Supplementary file2 (PDF 1879 KB) [file 705_2024_6081_MOESM2_ESM.pdf]

Figure S3

A

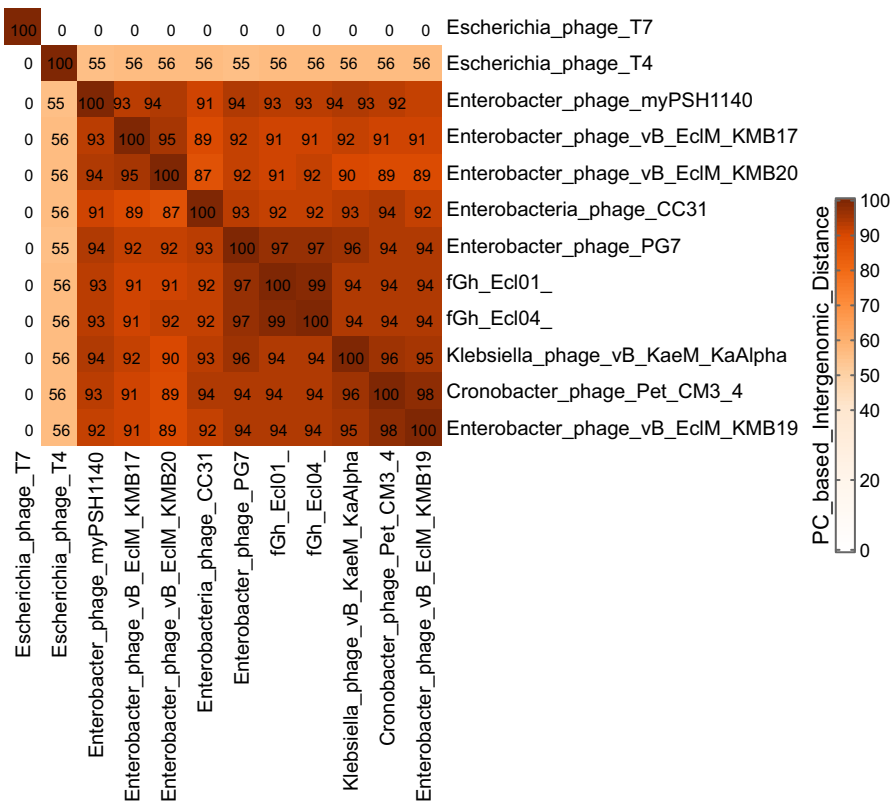

B

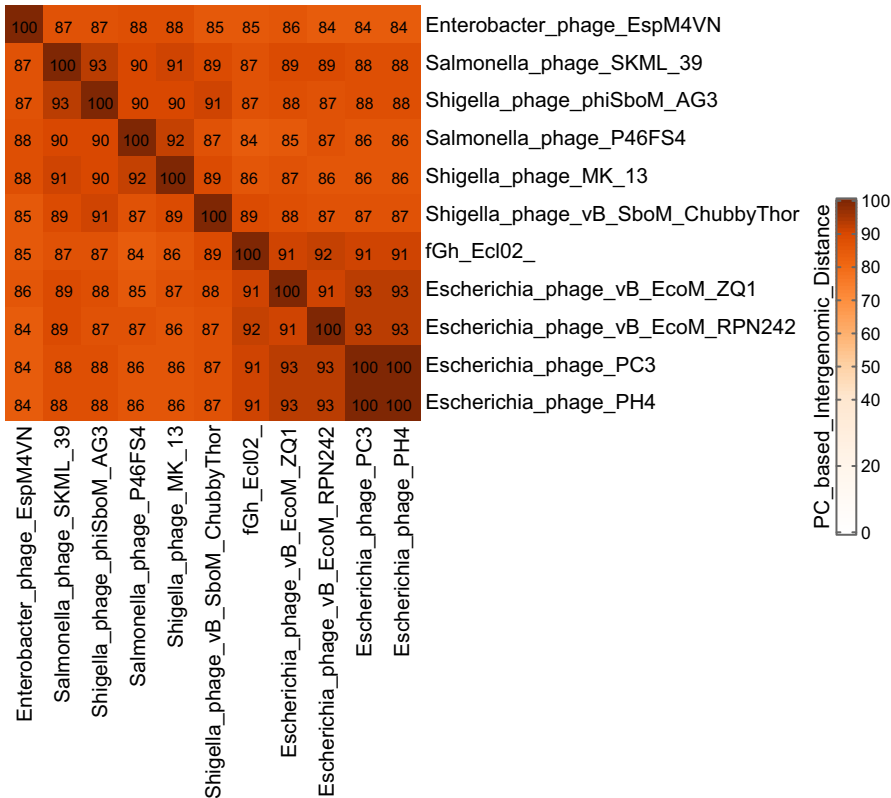

Supplement: Supplementary file 3 — Supplementary file3 (PDF 1361 KB) [file 705_2024_6081_MOESM3_ESM.pdf]
