# Supplementary material for: Three novel Enterobacter cloacae bacteriophages for therapeutic use from Ghanaian natural waters
Source: Arch Virol. 2024 Jul 5;169(8):156. doi: 10.1007/s00705-024-06081-9 (PMC11226500; doi:10.1007/s00705-024-06081-9)
Supplement: Supplementary file 4 — Supplementary file4 (PDF 20 KB) [file 705_2024_6081_MOESM4_ESM.pdf]

Table S1. Bacteriophage genomes chosen for VICTOR and VirClust analyses.

	Bacteriophage	Query coverage, %	Identity, %	Accession number
fGh-Ecl01	Enterobacter phage PG7	95	98.51	KJ101592.1
	Enterobacter phage vB-EclM_KMB19	95	94.94	OL828290.1
	Klebsiella phage vB_KaeM_KaAlpha	95	97.57	MN013084.1
	Cronobacter phage Pet-CM3-4	94	94.99	NC_055726.1
	Enterobacter phage myPSH1140	92	90.46	NC_055739.1
	Enterobacter phage vB-EclM_KMB17	91	91.97	OL849997.1
	Enterobacteria phage CC31	90	92.25	GU323318.1
	Enterobacter phage vB-EclM_KMB20	90	90.97	OL828291.1
fGh-Ecl02	Escherichia phage vB_EcoM-RPN242	87	92.23	OL656110.1
	Escherichia phage PH4	87	98.14	ON184126.1
	Escherichia phage PC3	87	98.14	ON184125.1
	Escherichia phage vB_EcoM-ZQ1	86	98.23	MW650886.1
	Shigella phage phiSboM-AG3	86	92.12	NC_013693.1
	Salmonella phage P46FS4	85	98.04	NC_049509.1
	Salmonella phage SKML-39	85	91.35	JX181829.1
	Shigella phage vB_SboS_Gloob	86	91.95	OL615011.1
	Shigella phage vB_SboM_ChubbyThor	85	93.42	OL615013.1
	Shigella phage MK-13	86	92.01	NC_049455.1
	Enterobacter phage EspM4VN	85	90.21	NC_049384.1
fGh-Ecl04	Enterobacter phage PG7	96	98.19	KJ101592.1
	Klebsiella phage vB_KaeM_KaAlpha	94	97.15	MN013084.1
	Enterobacter phage vB-EclM_KMB19	95	95.22	OL828290.1
	Enterobacteria phage CC31	89	92.24	GU323318.1
	Cronobacter phage Pet-CM3-4	95	95.32	NC_055726.1
	Enterobacter phage vB-EclM_KMB17	91	91.95	OL849997.1
	Enterobacter phage vB-EclM_KMB20	90	90.87	OL828291.1
	Enterobacter phage myPSH1140	92	90.31	NC_055739.1
