# Supplementary material for: Three novel Enterobacter cloacae bacteriophages for therapeutic use from Ghanaian natural waters
Source: Arch Virol. 2024 Jul 5;169(8):156. doi: 10.1007/s00705-024-06081-9 (PMC11226500; doi:10.1007/s00705-024-06081-9)
Supplement: Supplementary file 5 — Supplementary file5 (PDF 10 KB) [file 705_2024_6081_MOESM5_ESM.pdf]

Table S2. Tail fiber and spike proteins used for VICTOR analysis.

Bacteriophage	Protein annotation	Size, aa	Accession number
fGh-Ecl01	long tail fiber distal subunit	949	USL85756.1
fGh-Ecl02	tail spike protein	700	USL85810.1
fGh-Ecl04	long tail fiber distal subunit	949	USL86236.1
Enterobacter phage PG7	long tail fiber distal subunit	842	AHI61175.1
Enterobacter phage vB-EclM_KMB19	tail fibers protein	870	ULA52493.1
Klebsiella phage vB_KaeM_KaAlpha	hypothetical protein KAALPHA_299	871	QEG13317.1
Cronobacter phage Pet-CM3-4	Long tail fiber, distal subunit	871	YP_010091871.1
Enterobacter phage myPSH1140			
Enterobacter phage vB-EclM_KMB17	tail fibers protein	900	UKH49656.1
Enterobacteria phage CC31	gp37 long tail fiber, distal subunit	870	ADB81753.1
Enterobacter phage vB-EclM_KMB20	long tail fiber	916	ULA52771.1
Escherichia phage vB_EcoM-RPN242	tail fiber protein	849	UHS65532.1
Escherichia phage PH4	tail spike 3	849	URX65980.1
Escherichia phage PC3	tail spike 3	849	URX65789.1
Escherichia phage vB_EcoM-ZQ1	tail fibers protein	1040	QVW27043.1
Shigella phage phiSboM-AG3	hypothetical protein phiSboM-AG3_gp175	594	YP_003358662.1
Salmonella phage P46FS4	tail spike protein	159	YP_009889341.1
Salmonella phage SKML-39	hypothetical protein	712	AFU64345.1
Shigella phage vB_SboS_Gloob	putative tail spike protein	740	UHS65013.1
Shigella phage vB_SboM_ChubbyThor	putative tail protein	594	UGO47145.1
Shigella phage MK-13			
Enterobacter phage EspM4VN			
Escherichia phage T4	gp37 long tail fiber, distal subunit	1026	AAD42460.1
Enterobacteria phage T7	tail fiber protein	553	NP_042005.1
